# Real‐World Evidence of Upadacitinib Effectiveness in Moderate to Severe Atopic Dermatitis Patients in Saudi Arabia: A Retrospective Chart Review

**DOI:** 10.1155/drp/1238540

**Published:** 2026-07-15

**Authors:** Shawky E. L. Saaty, Hamza Salim Alshehri, Osama Alsharif, Azhar Alali, Mohamad Ibrahim Fatani, Abdulmajeed Alajlan, Shereen Hammad, Ibrahim Elnahal, Tharwat Hamad, Ali Anwar, Kamal Ahmed

**Affiliations:** ^1^ Dermatology Department, East Jeddah Hospital, Jeddah, Saudi Arabia; ^2^ Dermatology Department, Aseer Central Hospital, Aseer, Saudi Arabia; ^3^ Dermatology Department, KFGH Madinah, Madinah, Saudi Arabia; ^4^ Dermatology Department, Hera General Hospital, Makkah, Saudi Arabia; ^5^ Dermatology Department, College of Medicine, King Saud University, Riyadh, Saudi Arabia, ksu.edu.sa; ^6^ Intercontinental Medical Affairs Department, AbbVie Biopharmaceuticals GmbH, Dubai, UAE; ^7^ Saudi Medical Affairs Department, AbbVie Biopharmaceuticals GmbH, Riyadh, Saudi Arabia; ^8^ Saudi Medical Affairs Department, AbbVie Biopharmaceuticals GmbH, Jeddah, Saudi Arabia; ^9^ Dermatology Department, Mohammad Dossary Hospital, Al Khobar, Saudi Arabia

**Keywords:** atopic dermatitis, effectiveness, Janus kinase inhibitors, real world, safety, Saudi Arabia, upadacitinib

## Abstract

**Background:**

Upadacitinib is an oral, selective, and reversible Janus kinase inhibitor that has demonstrated sustained efficacy and safety over 140 weeks in clinical trials for treating atopic dermatitis (AD). We evaluated the real‐world effectiveness and impact on quality of life (QoL) of upadacitinib‐based treatment therapy in patients with moderate‐to‐severe AD in Saudi Arabia (SA).

**Methods:**

A retrospective chart review was conducted at six sites. Data were collected from medical records of patients with AD aged ≥ 12 years who were treated with upadacitinib for a period of six months.

**Results:**

This study included 109 AD patients, with 15.6% (17/109) being adolescents (aged 12–17) and 84.4% (92/109) being adults (aged 18 and older). The mean age was 30.9 years (57.8% males). Based on disease severity assessment by the Eczema Area and Severity Index (EASI) scoring system, severe AD was observed in 65.1% of the patients. At six months, 83.5% of the patients had an EASI score ≤ 7 and EASI‐75, EASI‐90, and EASI‐100 were achieved by 83.5%, 57.8%, and 12.8%, respectively. Dermatology life quality index (DLQI) 0/1 and pruritus Numerical Rating Scale (NRS) 0/1 at 6 months were achieved by 28.4% and 64.2%, respectively. Improvements were observed in the EASI, DLQI, and pruritus‐NRS scores from baseline to the last visit (*p* < 0.001). The frequency of outpatient visits and filled prescriptions among AD patients remained stable after upadacitinib initiation, with a median (range) of 2 (1–20) outpatient visits before and 2 (1–9) after 6 months and a median (range) of 2 (1–12) filled prescriptions before compared with 2 (0–14) after 6 months. Adverse events (AEs) were reported in 10 patients, with acne being the most commonly observed.

**Conclusion:**

Upadacitinib‐based treatment strategies showed an improvement in DLQI, EASI, and pruritus‐NRS scores in patients with moderate‐to‐severe AD in SA. No new safety signals were identified, reinforcing its favorable benefit‐risk profile in AD management.

## 1. Introduction

Atopic dermatitis (AD), also known as atopic eczema, is a chronic, relapsing, inflammatory skin disease characterized by eczematous lesions and intense pruritus [1, 2]. It is often, but not necessarily, associated with elevated serum IgE levels [2]. It affects up to 10% of the adults and approximately 15%–30% of the pediatrics, especially in families with other atopic diseases [3]. Compared with individuals without AD, adults with AD are more likely to experience concomitant gastrointestinal, rheumatological, and dermatological autoimmune diseases [4, 5]. They also have a higher risk of psychological distress, decreased self‐esteem, greater comorbidity, and an overall lower health‐related quality of life (QoL) [4, 5].

According to a systematic review of epidemiological studies, the incidence and prevalence of AD have increased over the past 20 years across Africa, Eastern Asia, Western Europe, and some regions of Northern Europe [6]. Between 2008 and 2012, the prevalence of AD ranged from 6.1% to 13% across different regions in Saudi Arabia (SA) [7]. A recent cross‐sectional study involving 222 participants found that 13.1% had AD in SA between January and May 2018 [8].

Moderate‐to‐severe AD in adults may be challenging to treat due to the risk/benefit profiles of systemic therapies such as corticosteroids and cyclosporine. There is a substantial unmet need for safe and more efficient treatments, especially for patients with inadequate responses to currently available medications.

Recently, several new targeted therapies have been approved for treating AD. Upadacitinib is an oral, selective, and reversible Janus kinase (JAK) inhibitor, in human cellular assays, preferentially inhibits signaling by JAK1 or JAK1/3 with functional selectivity over cytokine receptors that signal via pairs of JAK2, reducing the signaling of many mediators responsible for AD manifestations, such as pruritus and eczematous skin lesions [9, 10].

Upadacitinib has been approved in seven indications in SA, including rheumatoid arthritis, psoriatic arthritis, nonradiographic axial spondyloarthritis, ankylosing spondylitis (radiographic axial spondyloarthritis), ulcerative colitis, Crohn’s disease, and AD [11]. For AD, it is indicated for the treatment of adults and adolescents aged 12 years and older with moderate‐to‐severe AD who are candidates for systemic therapy [12]. The recommended dose for adults with moderate‐to‐severe AD is 15 mg or 30 mg once daily, based on individual patient presentation. However, 15 mg once daily is the recommended dose for patients ≥ 65 years of age and adolescents from 12 to 17 years weighing at least 30 kg [12].

Several Phase 3 trials have shown that upadacitinib at doses of 15 mg and 30 mg is more effective than a placebo in treating moderate‐to‐severe AD in both adolescents and adults without increased adverse events (AEs) or serious AEs leading to study drug discontinuation [13, 14]. Beyond Phase 3 randomized clinical trials, numerous real‐world studies have confirmed the effectiveness and safety of upadacitinib in routine clinical practice [15]. However, data from the Middle East, and particularly from SA, remain scarce, creating a gap in understanding the real‐world performance of upadacitinib 15 mg/30 mg use in this population; therefore, we aimed to assess the clinical effectiveness and patient‐reported QoL outcomes, such as healthcare resource utilization (HCRU) and AD‐related sick leaves for moderate‐to‐severe AD patients who received upadacitinib as a part of their treatment in SA.

## 2. Methods

### 2.1. Study Design and Settings

This study is a retrospective, observational, single‐arm, multicenter chart review analysis from June to December 2023, adhering to the Strengthening the Reporting of Observational Studies in Epidemiology (STROBE) guidelines [16].

This study was conducted in SA at six sites, including King Fahad General Hospital, Madinah, King Khalid University Hospital, Aseer Central Hospital, East Jeddah Hospital, Heraa General Hospital, and Mohamed Dossary Hospital.

### 2.2. Participants

The study included patients aged 12 years or older who received upadacitinib treatment (15 mg or 30 mg) for more than six months before the planned data collection period. Eligible patients were required to have a documented diagnosis of moderate‐to‐severe AD, confirmed by the treating physician. The assessment of disease severity was based on the physician’s clinical judgment at the time of treatment initiation, considering factors such as the extent and severity of skin lesions, intensity of pruritus, and impact on QoL. Additionally, patients needed to have complete medical records for at least 6 months after upadacitinib initiation and available medical records for HCRU for at least 6 months before the treatment initiation.

Patients who did not have at least 6 months of follow‐up data in the medical records upon data collection or who did not receive upadacitinib, according to the Saudi Food and Drug Authority (SFDA) label, were excluded from this study.

### 2.3. Variables

The primary endpoint of this study was the proportion of patients with an Eczema Area and Severity Index (EASI) score ≤ 7 at 6 months. Disease severity was categorized as “moderate,” “severe,” or “very severe” based on the EASI score, which ranges from 0 to 72. This categorization used the validated EASI score cutoffs defined in a practical guide: scores of 7.1–21.0 indicate moderate disease, 21.1–50.0 indicate severe disease, and scores > 50.0 indicate very severe disease [17]. Secondary endpoints included the proportion of patients with an EASI score ≤ 7 at 3 months; dermatology life quality index (DLQI) scores at 3 and 6 months; mean change in DLQI scores from baseline to 3 and 6 months; proportion of patients with DLQI 0/1 at 3 and 6 months among those with DLQI > 1 at baseline; mean change in EASI scores from baseline to 3 and 6 months; proportion of patients achieving EASI‐75/90/100 at baseline and at 3 and 6 months; Numerical Rating Scale (NRS) scores at 3 and 6 months; mean change in NRS scores from baseline to 3 and 6 months; proportion of patients achieving an improvement (reduction) in NRS ≥ 4 from baseline to 3 and 6 months among participants with NRS ≥ 4 at baseline; proportion of patients with NRS 0/1 at 3 and 6 months among those with NRS > 1 at baseline; frequency of HCRU—including emergency room visits, ambulatory visits, outpatient visits, and inpatient visits—at 6 months before and after upadacitinib initiation; length of hospitalization in case of hospital admission; and frequency of AD‐related sick leaves 6 months before and after upadacitinib initiation.

A post hoc analysis was conducted to evaluate the proportion of patients achieving Minimal Disease Activity (MDA) optimal target at 6 months, defined as the achievement of both EASI‐90 and a Peak Pruritus Numerical Rating Scale (PP‐NRS) score ≤ 1 [18].

### 2.4. Ethical Considerations

Ethical approval was obtained from the research committee of each participating site. At each center, the protocol was approved by the ethics committee (protocol number: H23‐107). This study was conducted in accordance with the principles laid down by the 18^th^ World Medical Assembly (Helsinki, 1964), including all subsequent amendments.

### 2.5. Sample Size

Based on previous Phase III clinical trials, Measure UP 1 and 2 post hoc analyses, the proportion of patients achieving EASI 0–7 (clear, almost clear, or mild) at 4 months was 60.5% and 74% for upadacitinib 15‐mg QD and 30‐mg QD doses, respectively [13]. Based on surveys and qualitative advice from prescribing physicians, we assumed using 15 mg in 2/3 of the patients and 1/3 in 30 mg as the initiation dose in the real‐world setting. Therefore, the weighted response rate was assumed to be 65%.

The statistical precision, as measured by half‐width (margin error), assumed 10%, and the maximum standard deviation (SD) of % 0–7 at Month 6 was 0.5, based on a 95% confidence interval (CI); the sample size required was 107 patients with AD considering that 10% of the included patients would have missing or insufficient data. As this was a retrospective database study, the final analysis was conducted when sufficient data for EASI at 6 months were accumulated. Randomized controlled trial (RCT) findings are usually discounted in real‐world studies; 65% of the patients achieving EASI 0–7 can be reduced to 55%, which does not impact the sample size conclusion as the variance is stable over this response rate range.

### 2.6. Statistical Analysis

Statistical analyses were performed using SPSS Version 28.0 or higher (IBM, SPSS Inc., USA). A significance level of 0.05 was applied for all statistical tests.

Descriptive statistics: Categorical variables were summarized as frequencies and percentages. Continuous variables were assessed for normality using the Shapiro–Wilk test. Normally distributed data were presented as mean ± SD, while nonnormally distributed data were reported as median and range.

Comparative analyses: For paired continuous variables, paired *t*‐tests were used for normally distributed data, and Wilcoxon signed‐rank tests were used for nonnormally distributed data. For independent variables, independent *t*‐tests were used for normally distributed data, and Mann–Whitney *U* tests were used for nonnormally distributed data. Categorical variables were compared using chi‐square or Fisher’s exact tests.

Multivariable modeling: Key target variables were evaluated using generalized linear mixed models at the primary endpoint, with missing data below 10%. Models included fixed effects for age, sex, baseline disease severity, concomitant medication use, and upadacitinib dose, with study site specified as a random effect.

Missing data: A complete case analysis was performed, as the percentage of missing data was below 10% for key outcomes.

Notably, comparisons between 15 mg and 30 mg were neither primary nor secondary endpoint, and the study was not designed to evaluate differences in efficacy or safety between doses. Therefore, any dose‐related findings are exploratory and descriptive rather than definitive.

## 3. Results

### 3.1. Participants

A total of 109 eligible patients with AD were enrolled in the study from the six participating sites, and all were included in the effectiveness and safety analysis.

### 3.2. Descriptive Statistics

#### 3.2.1. Sociodemographic Characteristics

In a study of 109 individuals, 15.6% (17/109) were adolescents (aged 12–17), while the remaining 84.4% (92/109) were adults (aged 18 and older). Among the participants in the study, 63 (57.8%) were males and 94 (86.2%) were of Saudi nationality. Of these, 46.8% of the patients were Arab, 27 (24.8%) Asians, 16 (14.7%) Caucasian, and 1.8% Black or African American, while ethnicity data were not available for 11.9% of the patients. Furthermore, 40 (36.7%) had full‐time employment, while 33 (30.3%) were still studying. The average population age (SD) was 30.9 (13.5) years. Only 10 (9.2%) of the patients reported tobacco/nicotine use (Table 1).

**TABLE 1:** Baseline demographic characteristics of study participants (*n* = 109).

Variable	*N* (%) or mean ± SD
*Gender*	
Male	63 (57.8%)
Female	46 (42.2%)

*Age (years)*	30.9 ± 13.5

*Nationality*	
Saudi	94 (86.2%)
Non‐Saudi	14 (12.8%)
NA	1 (0.9%)

*Race*	
Arab	51 (46.8%)
Asian	27 (24.8%)
Caucasian	16 (14.7%)
Black or African American	2 (1.8%)
NA	13 (11.9%)

*Tobacco/nicotine use*	
User	10 (9.2%)
Nonuser	69 (63.3%)
NA	30 (27.5%)

*Employment status*	
Not yet (studying)	33 (30.3%)
Full time	40 (36.7%)
Part‐time	3 (2.8%)
Unemployed	12 (11%)
Retired	6 (5.5%)
NA	15 (13.8%)

*Note:* Other category primarily represents Middle Eastern ethnicity, consistent with the predominant population demographics in Saudi Arabia.

Abbreviation: NA, not available.

#### 3.2.2. Medical and Comorbid Conditions

Of the reported associated comorbidities, the most common conditions were asthma (21.2%), hypertension (15.2%), diabetes (12.1%), allergic rhinitis (12.1%), and allergic conjunctivitis (12.1%). Moreover, 42 patients (38.5%) reported a positive family history of AD (Table 2). At baseline, there was only one patient with a previous medical history of acne.

**TABLE 2:** Comorbidities among study participants.

Variable	*N* (%)
*Comorbidities* ^∗^	
Asthma	7 (21.2%)
Allergic rhinitis	4 (12.1%)
Chronic sinusitis	1 (3%)
Allergic conjunctivitis	4 (12.1%)
Depression	1 (3%)
Food allergy	2 (6.1%)
Diabetes mellitus	4 (12.1%)
Hypertension	5 (15.2%)
Other	5 (15.2%)

*The family history of atopic dermatitis*	
Yes	42 (38.5%)
No	41 (37.6%)
NA	26 (23.9%)

Abbreviation: NA, not available.

^∗^Patient might have more than one comorbidity.

#### 3.2.3. AD and Upadacitinib‐Related Characteristics

The median duration (range) of AD among the population was 33 months (8–537). Based on the disease severity assessment using the EASI scoring system, 71 (65.1%) and 38 (34.9%) patients had severe and moderate AD at the baseline, respectively. The median (range) percent of the affected body surface area (BSA) at diagnosis was 40 (12–85), and its morphological patterns involved both extremities (25.7%), trunk (24.3%), flexural areas (20.8%), and the face (14.4%), while at the baseline visit, the median (range) percent of the affected BSA was 35 (10–90) (Table 3).

**TABLE 3:** Baseline atopic dermatitis and upadacitinib‐related characteristics (*n* = 109).

Variables	Values
*Disease-related information*	
AD disease duration (months), median (range)	33 **(**8–537)
The severity of atopic dermatitis, *N* (%)	
Moderate	38 (34.9%)
Severe	71 (65.1%)
Percent of BSA affected, median (range)	35 (10–90)
Morphological pattern of the affected BSA^∗^, *N* (%)	
Flexural	84 (20.8%)
Face	58 (14.4%)
Extremity	104 (25.7%)
Trunk	98 (24.3%)
Hand	41 (10.1%)
Other	19 (4.7%)

*Upadacitinib information*	
The prescribed dose of upadacitinib, *N* (%)	
15 mg	98 (89.9%)
30 mg	11 (10.1%)
Reason for 30‐mg dose selection, *N* (%)	
Mild improvement in the patient’s condition	1 (9%)
Severity of disease	10 (91%)

Abbreviations: AD, atopic dermatitis; BSA, body surface area.

^∗^Patient might have more than one morphological pattern.

Among the study population, 98 (89.9%) patients received 15 mg and 11 (10.1%) received 30 mg of upadacitinib. The decision to start the high dose was influenced mainly by the severity of the disease, as assessed by the EASI score (91%) (Table 3).

#### 3.2.4. Concomitant Medications

##### 3.2.4.1. Concomitant AD Medications

The number of patients previously prescribed medications for AD decreased from 90 (82.6%) at the baseline to 20 (18.3%) after six months of upadacitinib treatment. At baseline, 25 patients (23%) received topical treatments while 84 patients (77%) received systemic treatments. At the 6‐month visit, the number of patients receiving topical treatments decreased to 13 (12%) and the number receiving systemic treatments increased to 96 (88%). Initially, the most reported medications were cyclosporine (16.8%), prednisolone (15%), and betamethasone (10.5%). After 3 months, mometasone (24.5%) and cetirizine (15.1%) were the most reported prescribed medications. At the 6‐month visit, mometasone (15.2%) and cetirizine (12.1%) remained the most frequently reported medications (Table Supporting 1).

Of the patients taking upadacitinib, 77.1% were using other AD medications at the start of the study, slightly dropping to 75.2% at the 6‐month mark. Among them, 72.5% were receiving other topical AD medications, which dropped to 68.8% by the 6‐month visit, while 27.5% were receiving other systemic treatments, which increased to 34.3% at the 6‐month visit. Cetirizine, mometasone, and white paraffin were the primary AD medications used initially and continued to be the most common choices at both three and six months (Supporting file, Table 1).

##### 3.2.4.2. Concomitant Non‐AD Medications

Concomitant medications for conditions other than AD were prescribed for 18 patients (16.5%) at baseline, decreasing slightly to 16 patients (14.7%) after three months and 17 patients (15.6%) after six months. At baseline, salbutamol, ferrous sulfate, and levothyroxine were each prescribed to 2 patients (6%). Also, salbutamol and levothyroxine were most frequently prescribed at 3 months (5.7% for each of them). After 6 months, adapalene became the most frequently prescribed medication, used by 10.8% of the patients (Supporting file, Table 2).

Asthma and hypertension were the most common indications for concomitant non‐AD medications at baseline (14.7% and 14.7%, respectively) and after 3 months (14.3% and 17.1%, respectively). However, after 6 months, acne became the most frequent indication (18.9%), followed by hypertension (16.2%) and bronchial asthma (13.5%). The number of cases receiving medications for acne increased from 1 (2.9%) at baseline to 4 (11.4%) at 3 months and 7 (18.9%) at 6 months. After exclusion of acne as an indication for concomitant medications, 21 (65.6%) were still receiving their concomitant medications at baseline. This figure increased to 23 patients (65.7%) after three months and 27 patients (72.9%) after six months **(**Table Supporting 2).

#### 3.2.5. Upadacitinib Prescription Patterns

During the first visit (Month 3), 108 patients (99.1%) continued to receive upadacitinib. This number declined on the second visit (Month 6) to 99 patients (90.8%). Since only 10.1% of the patients received the 30 mg dose, dose‐related observations are described solely descriptively. At 3 and 6 months, we observed that 15 and 6 patients, respectively, required dose escalation from 15 mg to 30 mg. Conversely, de‐escalation from 30 mg to 15 mg was needed for 3 patients at 3 months and for 4 patients at 6 months (Table 4). The main reason for escalation was the inadequate response (defined as not achieving EASI‐75), which occurred in six patients (40%) on the first visit and in two patients (33.3%) on the second visit. However, the reason for de‐escalation was the favorable clinical response (defined as achieving EASI‐75) of 100% on the first visit and 50% on the second visit. Nonadherence was the cause of drug interruption during the first visit, which was reported only in one patient. However, the unavailability of the drug was the main reason for drug interruption at the second visit, which was reported in three patients. The median duration of interruption was longer on the second visit (31.5 days) compared with 14 days on the first visit. Moreover, the main reasons for drug discontinuation reported on the second visit were the lack of response or AEs, which were reported by two patients in each. One patient’s discontinuation was temporary, as treatment was resumed after the AE resolved. For the other patient, the treatment for the AE is still ongoing and the drug was permanently discontinued till the end of the study period (Table 4).

**TABLE 4:** Upadacitinib prescription patterns.

Variable	Visit 1 (Month 3)	Visit 2 (Month 6)
*Treatment status, N (%)*		
Continued	108 (99.1%)	99 (90.8%)
Interrupted	1 (0.9%)	4 (3.7%)

*Usage pattern (in case of continued treatment), N (%)*		
Escalation from 15 mg to 30 mg	15 (13.9%)	6 (6.1%)
De‐escalation from 30 mg to 15 mg	3 (2.8%)	4 (4%)
The same previously prescribed dose	90 (83.3%)	89 (89.9%)

*Reason for escalation, N (%)*		
Attacks of itching	0	1 (16.7%)
Frequent flares	1 (6.7%)	0
Inadequate effect	3 (20%)	1 (16.7%)
Inadequate response	6 (40%)	2 (33.3%)
Partial remission	1 (6.7%)	0
Poor response	4 (26.6%)	1 (16.7%)
Suboptimal improvement	0	1 (16.7%)

*Reason for de-escalation, N (%)*		
Favorable clinical response	3 (100%)	2 (50%)
The recommended dose in the MOH is no more than 15 mg	0	1 (25%)
Severity response and improvement	0	1 (25%)

*Cause of interruption, N (%)*		
Noncompliance	1 (100%)	0
No improvement	0	1 (25%)
Unavailability of drug	0	3 (75%)

Duration of interruption (days), median (range)	14	31.5 (20–34)

Reason for discontinuation*^∗^*, N (%)		
Drug availability/reimbursement	NA	1 (12.5%)
Lack of response	NA	2 (25%)
Adverse event experience	NA	2 (25%)
Disease progression	NA	1 (12.5%)
Other	NA	2 (25%)

*Discontinuation due to adverse events*		
Recovered and continue on upadacitinib	NA	1 (12.5%)
Treatment of the adverse event is ongoing	NA	1 (12.5%)

Abbreviations: MOH, ministry of health; NA, not available.

^∗^Patient might have more than one reason.

### 3.3. Main Results

#### 3.3.1. EASI Score

**FIGURE 1:**
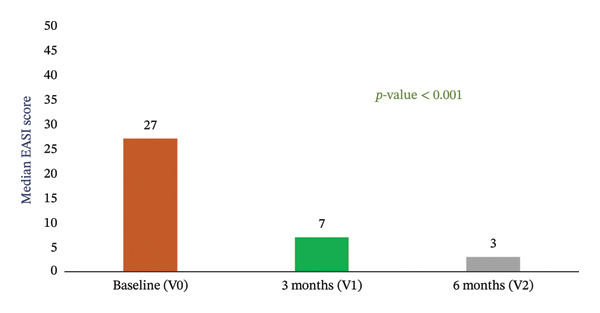
Median change in EASI score (ranges from 0 to 72) from baseline to Month 3 and Month 6 (*n* = 109).

The median (range) EASI decreased from 27 (8–60) at baseline to 7 (1–40) after three months and then to 3 (0–30) after six months (*p* < 0.001). The data revealed that 31 (28.4%), 56 (51.4%), and 22 (20.2%) patients had moderate, severe, and very severe disease at the baseline. After 3 months, the majority of patients had mild (53.2%) or moderate (39.4%) disease, and none had a very severe disease. After the second visit, 76 (69.7%) patients had a mild disease, while none had a very severe disease (Table 5) (Figure 1).

**TABLE 5:** Effectiveness and quality of life endpoints after 6 months’ treatment with upadacitinib (*n* = 109).

Endpoint	Baseline	Visit 1 (Month 3)	Visit 2 (Month 6)
*EASI score*			
EASI, median (range)	27 (8–60)	7 (1–40)	3 (0–30)
Change in EASI score from baseline			
MD (95% CI)	NA	20.9 (18.6–23.2)	26.3 (23.5–29.1)
*p* value^∗^	NA	< 0.001	< 0.001
Interpretation of EASI score, *N* (%)			
Clear	0 (0%)	0 (0%)	14 (12.8%)
Mild	0 (0%)	58 (53.2%)	76 (69.7%)
Moderate	31 (28.4%)	43 (39.4%)	15 (13.8%)
Severe	56 (51.4%)	8 (7.3%)	4 (3.7%)
Very severe	22 (20.2%)	0 (0%)	0 (0%)
EASI ≤ 7, *N* (%)	NA	61 (56%)	91 (83.5%)
EASI‐75, *N* (%)	NA	55 (50.5%)	91 (83.5%)
EASI‐90, *N* (%)	NA	2 (1.8%)	63 (57.8%)
EASI‐100, *N* (%)	NA	0 (0%)	14 (12.8%)

*DLQI*			
DLQI, median (range)	17 (5–30)	5 (0–20)	2 (0–22)
Change in DLQI score from baseline			
MD (95% CI)	NA	10.1 (9.2–11)	13 (11.9–14)
*p* value^∗^	NA	< 0.001	< 0.001
Interpretation of DLQI score, *N* (%)			
No effect at all on patient’s life	0 (0%)	2 (1.8%)	29 (26.6%)
Small effect on patient’s life	1 (0.9%)	59 (54.1%)	57 (52.3%)
Moderate effect on patient’s life	19 (17.4%)	31 (28.4%)	16 (14.7%)
Very large effect on patient’s life	59 (54.1%)	17 (15.6%)	6 (5.5%)
Extremely large effect on patient’s life	30 (27.6%)	0 (0%)	1 (0.9%)
DLQI 0/1, *N* (%)	NA	2 (1.8%)	31 (28.4%)

*NRS*			
NRS, median (range)	8 (3–10)	3 (0–8)	1 (0–9)
Change in NRS score from baseline			
MD (95% CI)	NA	4.7 (4.3–5.1)	5.9 (5.6–6.4)
*p* value^∗^	NA	< 0.001	< 0.001
NRS score improvement^∗∗^ (n = 107), *N* (%)			
Reduction equal to or more than 4	NA	88 (82.2%)	96 (89.7%)
Reduction of less than 4	NA	19 (17.8%)	11 (10.3%)
Pruritus NRS, *N* (%)			
Pruritus NRS 0/1	NA	9 (8.3%)	70 (64.2%)
Pruritus NRS score more than 1	NA	100 (91.7%)	40 (36.7%)

*BSA*			
Percent of the affected BSA, median (range)	35 (10–90)	10 (0–60)	7 (0–50)

*MDA optimal target (EASI-90+ PP-NRS ≤ 1)*			
*N* (%)	NA	NA	53 (48.6%)

*HCRU (6 months before upadacitinib initiation and 6 months after upadacitinib initiation)*			
Number of emergency room visits, *N* (%)			
No visits	76 (69.7%)	NA	75 (68.8%)
One visit	1 (0.9%)	NA	0 (0%)
NA	32 (29.4%)	NA	34 (31.2%)
Number of ambulatory visits, *N* (%)			
No visits	76 (69.7%)	NA	74 (67.9%)
3 visits	0 (0%)	NA	1 (0.9%)
NA	33 (30.3%)	NA	34 (31.2%)
Number of inpatient visits, *N* (%)			
No visits	76 (69.7%)	NA	75 (68.8%)
NA	33 (30.3%)	NA	34 (31.2%)
Number of outpatient visits, median (range)	2 (1–20)	NA	2 (1–9)
Change in outpatient visits from baseline			
SMD (95% CI)	NA	NA	2.7 (−0.13–0.94)
*p* value^∗^	NA	NA	0.343
Number of prescriptions filled, median (range)	2 (1–12)	NA	2 (0–14)
Change in prescriptions filled from baseline			
SMD (95% CI)	NA	NA	1.3 (−0.12–0.43)
*p* value^∗^	NA	NA	0.353

*Sick leaves*			
Sick leaves due to AD condition, *N* (%)			
Yes	6 (5.5%)	NA	2 (1.8%)
No	93 (85.3%)	NA	92 (84.4%)
NA	10 (9.2%)	NA	15 (13.8%)
Number of sick leaves due to AD condition (times), median (range)	2 (1–5)	NA	2.5 (2–3)
Duration of sick leaves due to AD condition (in days), median (range)	6.5 (1–22)	NA	10.5 (6–15)

Abbreviations: AD, atopic dermatitis; BSA, body surface area; DLQI, Dermatology Life Quality Index; EASI, Eczema Area and Severity Index; HCRU, healthcare resource utilization; NA, Not Available; NRS, Numerical Rating Scale; SMD, standardized mean differences.

^∗^Wilcoxon signed‐ranks test.

^∗∗^Two patients had NRS scores less than 4 at baseline.

**FIGURE 2:**
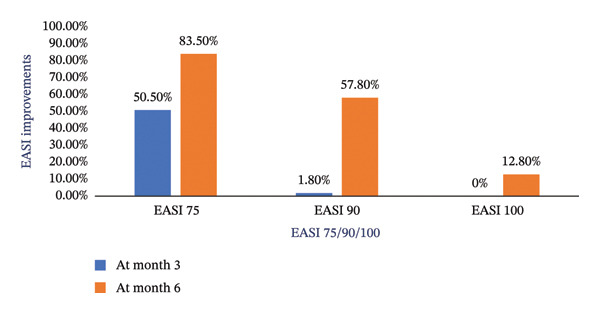
The proportion of patients achieving EASI‐75/90/100 from baseline at Month 3 and Month 6 (*n* = 109).

The proportion of AD patients with EASI ≤ 7 was 56% at 3 months, increasing to 83.5% at 6 months. Additionally, the proportion of patients with EASI‐75 was 50.5% and 83.5% at three and six months, respectively. Moreover, the proportions of participants who achieved an EASI‐90 score were 1.8% and 57.8% at three and six months, respectively. EASI‐100 was not reported at three months; however, this was achieved in 12.8% at six months (Table 5) (Figure 2).

#### 3.3.2. DLQI

**FIGURE 3:**
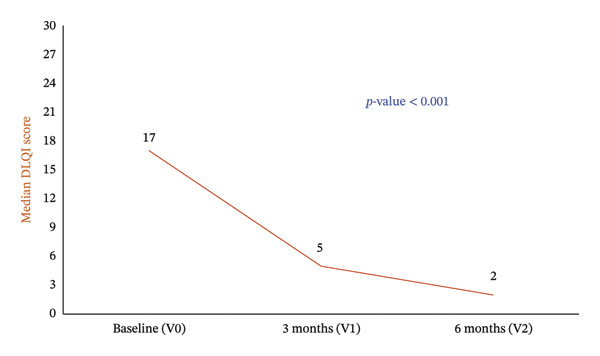
Median change in DLQI (ranges from 0 to 30) from baseline to Month 3 and Month 6 (*n* = 109).

The median (range) DLQI decreased from 17 (5–30) at baseline to 5 (0–20) after three months and 2 (0–22) after six months (*p* < 0.001). When interpreting the DLQI, at baseline, the disease had a very large effect on the lives of 59 (54.1%) patients. After three months, the disease had a small effect on the lives of 59 (54.1%) patients and a moderate effect on 31 (28.4%) patients. After six months, the disease had a small effect on the lives of 57 (52.3%) patients, and only one patient had an extremely large effect on his life. The proportion of AD patients with DLQI 0/1 at 3 and 6 months was 1.8% and 28.4%, respectively. The full data of DLQI are presented in Table 5 and Figure 3.

#### 3.3.3. NRS

**FIGURE 4:**
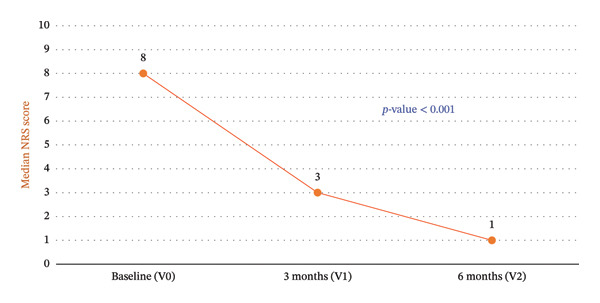
Median change in itch pruritus‐Numerical Rating Scale (NRS) score (ranges from 0 to 10) from baseline to Month 3 and Month 6 (*n* = 109).

The median (range) NRS score for pruritus was 8 (3–10) at baseline, which decreased to 3 (0–8) after 3 months, with a *p* value < 0.001. After 6 months, the score decreased to 1 (0–9) with a *p* < 0.001. The percentage of AD patients achieving NRS score reduction ≥ 4 from baseline was 82.2% and 89.7% at three and six months, respectively. The percentage of participants who achieved pruritus NRS 0/1 was 8.3% and 64.2% at three and six months, respectively (Table 5) (Figure 4).

#### 3.3.4. BSA

The median (Range) percent of the affected BSA was reduced from 35 (10–90) at the baseline to 10 (0–60) and 7 (0–50) after three and six months, respectively (Table 5).

#### 3.3.5. MDA Optimal Target

**FIGURE 5:**
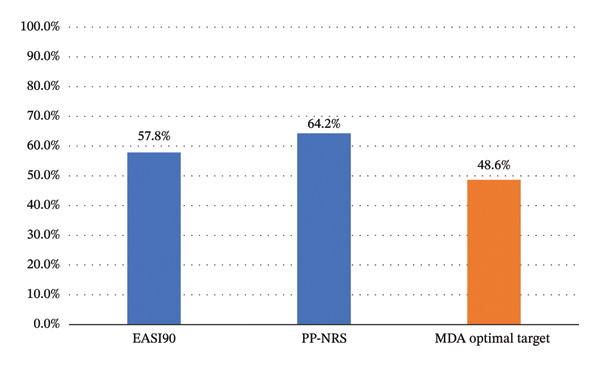
The proportion of patients achieving EASI‐90, PP‐NRS ≤ 1, and MDA optimal target at Month 6 (*n* = 109).

The proportion of patients achieving MDA optimal target, defined as the achievement of both EASI‐90 and PP‐NRS ≤ 1, at 6 months was 53 (48.6%) (Table 5) (Figure 5).

#### 3.3.6. HCRU

The frequency of outpatient visits and filled prescriptions among AD patients remained stable after upadacitinib initiation, with a median (range) of 2 (1–20) outpatient visits before and 2 (1–9) after 6 months and a median (range) of 2 (1–12) filled prescriptions before compared with 2 (0–14) after 6 months (Table 5).

#### 3.3.7. Sick Leaves

The median (range) of sick leaves related to AD was 2 (1–5) and 2.5 (2–3) at 6 months before and after upadacitinib initiation, respectively (Table 5).

### 3.4. AEs and Adverse Drug Reactions

Among the included 109 patients, a total of 10 AEs were recorded, four of which were cases of acne. Acne cases were graded as 3 mild and 1 moderate, all managed with topical retinoids (adapalene in 3 cases), and none required treatment discontinuation. All AEs were possibly related to the study medication. Nine of the AEs were either fully recovered or still recovering. Also, five AEs were evaluated as mild, four were moderate, only one was severe, and none of the reported AEs were considered serious. Upadacitinib was discontinued due to AEs in five cases, while no intervention was initiated for the remaining AEs. Corrective treatments were employed in six patients (60%) to address the reported AEs and included adapalene (4 cases), benzoyl peroxide, clindamycin, fusidic acid, and amoxicillin–clavulanic acid (1 case each) (Table 6). The safety profile of upadacitinib was consistent with the known safety profile reported in global studies, with no new safety signals identified [19].

**TABLE 6:** Summary of treatment‐emergent adverse events.

Adverse events	*N* (%)
*Experience any adverse events (n = 109)*	
Visit 1 (Month 3)	5 (4.6%)
Visit 2 (Month 6)	6 (5.5%)

*Adverse events (n = 10)*	
Acne	4 (40%)
Depression	1 (10%)
Dyspnea	1 (10%)
Hyperlipidemia	1 (10%)
Nodulocystic Acne	1 (10%)
Recurrent acne, boils and furunculosis	1 (10%)
Recurrent sore throat infection	1 (10%)

*Outcome (n = 10)*	
Recovered	6 (60%)
Recovering	3 (30%)
Not recovered	1 (10%)

*Severity (n = 10)*	
Mild	5 (50%)
Moderate	4 (40%)
Severe	1 (10%)

*Relationship to study medication (n = 10)*	
Reasonable possibility	10 (100%)

*Action taken with study medication (n = 10)*	
No action taken	4 (40%)
Dose increased	1 (10%)
Treatment discontinued	5 (50%)

*Corrective treatment (n = 10)*	
Yes	6 (60%)
No	4 (40%)

Corrective treatment*^∗^* (n = 6)	
Adapalene	4 (50%)
Amoxicillin + clavulanic acid	1 (12.5%)
Benzoyl peroxide	1 (12.5%)
Clindamycin	1 (12.5%)
Fusidic acid	1 (12.5%)

*Seriousness (n = 10)*	
Nonserious	10 (100%)

^∗^Patient might receive more than one corrective medication.

## 4. Discussion

The current study provides insights into the real‐world effectiveness of upadacitinib among moderate‐to‐severe AD patients in SA. This study revealed that upadacitinib‐based treatment strategies may improve clinician‐ and patient‐reported outcomes among patients with moderate‐to‐severe AD. We observed that 83.5% of the participants achieved an EASI score ≤ 7 at 6 months.

Furthermore, the proportion of AD patients achieving EASI‐75, EASI‐90, DLQI 0/1, and NRS‐pruritus ≥ 4 after three months of treatment was 50.5%, 1.8%, 1.8%, and 82.2%, respectively. After 6 months of treatment, these proportions further improved to 83.5%, 57.8%, 28.4%, and 89.7%, respectively. Furthermore, the difference in median EASI, DLQI, and NRS scores between the baseline and after three and six months of treatment with upadacitinib showed marked improvements (*p* < 0.001 for each). The results are consistent with the upadacitinib Phase 3 program, with an exception made for the results related to the proportion of patients achieving EASI‐90 at month 3 (1.8% at 3 months vs. 57.8% at 6 months): in the Phase 3 program at 3 months, around 15% of the patients achieved EASI‐90. This discrepancy may be attributed to missing data or absence of standardized assessment protocols, different population vs. Phase 3 program; furthermore, the threshold for marking a patient as. EASI‐90/100 in clinical practice may differ from trials with established rules for EASI. Finally, this study has a high proportion of patients with severe or very severe disease, which can account for a challenge in achieving a more stringent target [20, 21].

Our observation of the dose escalation pattern in our study population aligns with recent real‐world evidence on upadacitinib dosing strategies. We observed that 13.9% of the patients at 3 months and 6.1% at 6 months required dose escalation from 15 mg to 30 mg due to inadequate response. A real‐world study of including patients with insufficient response to 15 mg demonstrated marked improvements following dose escalation, with EASI‐75 achievement increasing from 4.3% to 68.2% within 4 weeks and EASI‐90 rising from 0% to 38.1% at 12 weeks postescalation. These findings support dose escalation to 30 mg as an effective strategy for patients not achieving adequate control on 15 mg [22]. The proportion of patients who achieved EASI‐75 in this study (83.5% at six months) was higher than what was reported in previous large clinical trials such as Measure Up 1 (74.7% at four months), Measure Up 2 (66.7% at four months), AD Up (70.9% at four months), and Heads Up (72.4% four months) [13, 14, 23, 24]. These results align with recent real‐world evidence showing the sustained effectiveness of upadacitinib over 52 weeks, with EASI 75, EASI 90, and EASI 100 achieved by 88.9%, 70.8%, and 54.2% of the patients, respectively. The comparable or slightly higher response rates observed in real‐world settings might be attributed to the flexibility in the use of concomitant therapies and dose optimization strategies in routine clinical practice [25].These findings suggest that the efficacy of upadacitinib is sustained over longer follow‐up periods. However, the proportion of AD patients achieving EASI‐100 was lower than that reported in the clinical studies; clarifications to this may be similar to what is described for EASI‐90 results [13, 14, 23, 24].

Compared with a prospective cohort study by Boesjes et al., a higher proportion of patients in this study treated with upadacitinib achieved EASI‐75, with 83.5% versus 73%, respectively. This discrepancy might be attributable to the lower EASI scores observed in the Boesjes et al. study compared with ours and the different follow‐up periods [26].

Another study conducted by Napolitano et al. demonstrated a comparable reduction in AD outcomes (pruritus NRS, EASI, and DLQI) to this study [27]. However, the follow‐up period of this study was only 16 weeks, compared with a maximum of six months (24 weeks) in this study [27]. Better clinical responses in terms of EASI‐75, EASI‐90, EASI‐100, EASI ≤ 7, and pruritus NRS score ≤ 4 were reported after 16 weeks of upadacitinib treatment in studies conducted by Gargiulo et al. and Chiricozzi et al. [28, 29]. This may be attributed to the small sample size and the inclusion of difficult‐to‐treat patients, such as those with several comorbidities or those who had previously failed other lines of therapies and had no other valid treatment options.

After three and six months of treatment in this study, we observed that 13.9% and 6.1% of the patients required dose escalation due to inadequate response. Increasing the upadacitinib dose from 15 to 30 mg alleviated pruritus and improved the EASI‐75 and EASI‐90 in a previous real‐world clinical practice in Japan [22]. Recent evidence supports dose escalation for those who are not responding to the 15‐mg dose. In a study of 31 patients receiving upadacitinib at 30 mg for 52 weeks, 84% achieved EASI‐90% and 68% achieved EASI‐100 (complete skin clearance), with significant improvements in itch and sleep outcomes [30]. Thus, dose escalation to 30 mg of upadacitinib could be considered for AD patients who do not respond sufficiently to 15 mg of upadacitinib. Furthermore, in a large Italian multicenter study, dose adjustments were made for patients based on treatment responses. Specifically, the dose was increased from 15 mg to 30 mg for 31 patients who experienced insufficient efficacy. Conversely, 23 patients had their dose reduced from 30 mg to 15 mg, 12 due to clinical improvement and 11 due to AEs. This emphasizes the importance of dose flexibility and personalized management [31].

Identifying patients who will benefit from long‐term treatment is crucial. A study evaluated predictive factors for long‐term high responders (IGA 0/1 at week 48) among 94 patients. For the 15‐mg dose, long‐term responders exhibited lower rates of bronchial asthma, lower baseline head/neck EASI scores, lower serum IgE levels, and lower systemic inflammatory response index (SIRI) values. For the 30‐mg dose, the key predictor was lower baseline IgE. These findings indicate that patients with a reduced Type‐2 inflammatory burden may respond better to JAK inhibition, which can aid in optimizing patient selection and dosing strategies [32]. Moreover, continuous treatment with upadacitinib was recommended to maintain skin clearance and the antipruritic effects [33].

Here, the frequency of outpatient visits, filled prescriptions, and sick leaves remained stable after initiation of upadacitinib. The safety profile of upadacitinib in the present study was favorable, with few AEs reported during the study period. Acne was the most frequently observed AE among the study participants, and it was evaluated as mild or moderate and clinically manageable. Acne was previously reported to affect 10% of the patients with moderate‐to‐severe AD treated with upadacitinib [34]. A previous study reported that patients treated with the 30‐mg dose had higher rates of acne than those on the 15‐mg dose [35]. The safety profile of upadacitinib is consistent with the known safety profile reported in global studies, with no new safety signals identified.

Beyond commonly recognized AEs, recent real‐world studies have identified additional JAK inhibitor–associated effects that warrant monitoring. Weight gain has emerged as an under‐recognized AE with JAK inhibitors, particularly those with JAK1/2 activity. A systematic review of 16,000 patients found that 5.9% experienced weight gain with JAK inhibitor therapy, with higher rates (7%) observed in RCTs. Another study comparing selective JAK1 versus JAK1/2 inhibitors demonstrated that patients receiving JAK1/2 inhibitors showed greater weight gain over 52 weeks compared with those on JAK1‐selective agents, likely due to impaired leptin signaling. In our study, weight changes were not systematically documented in the retrospective chart review. However, one case of hyperlipidemia was identified, which may be metabolically related to weight changes. Future prospective studies should include systematic monitoring of weight and metabolic parameters to better characterize this emerging safety concern with upadacitinib therapy [36, 37].

Despite the major differences in treatment performance between real‐world practice and clinical trials, we observed a comparable effectiveness of upadacitinib in clinical practice with previous clinical trials. The improved therapeutic effectiveness rates could be attributed to the concomitant application of corticosteroids when needed.

The high rate of concomitant therapy use (77.1% at baseline, 75.2% at 6 months) may have confounded our results. Notably, 68.8% of the patients continued topical corticosteroids at 6 months, and some received systemic corticosteroids. These background therapies likely contributed to the observed improvements in EASI, DLQI, and pruritus scores, limiting our ability to attribute outcomes solely to upadacitinib. This combination approach reflects real‐world practice but may overestimate upadacitinib’s effectiveness as monotherapy.

This study has some limitations that need to be considered. First, the retrospective design, short follow‐up period, the relatively small sample size, and the presence of some missing data may limit the generalizability of the findings. Second, the cohort was weighted toward patients with severe AD, which may reflect real‐world clinical practice in tertiary dermatology centers but might not represent the broader AD population in SA. Third, the study lacked a control group, which limits the ability to draw causal inferences. Fourth, data on prior JAK inhibitors used in AD were unavailable. Fifth, the outcomes of switching to 30 mg of upadacitinib should be further examined. Sixth, clinical phenotypes of AD were not explored. Finally, the concomitant use of corticosteroids and immunosuppressive drugs at baseline and throughout the study period may have affected our findings and introduced bias. These concomitant treatments likely contributed substantially to the observed improvements in EASI, DLQI, and pruritus‐NRS scores. As a result, it is difficult to separate the individual impact of conventional systemic treatments from that of upadacitinib on the noted improvements. Furthermore, the limited number of patients receiving specific systemic corticosteroids or immunosuppressants at each visit (typically < 10 per subgroup) precluded meaningful stratified or sensitivity analyses to isolate the effect of upadacitinib from concomitant therapies. Therefore, the reported effectiveness should be understood as the result of a comprehensive treatment strategy in which upadacitinib was an essential element rather than as the effect of upadacitinib monotherapy. Despite these limitations, the results reflect real‐world clinical practice. Although the study sponsor (AbbVie) was involved in the study design, data collection, analysis, and manuscript preparation, potential bias was mitigated by ensuring that data collection was performed by independent clinical investigators, the study was executed by an independent organization, and all authors had full access to the data and contributed to its interpretation.

## 5. Conclusion

Upadacitinib‐based treatment strategies showed sustained improvement in AD signs and symptoms and in patient‐related outcomes, including pruritus, sleep quality, and health‐related QOL, as measured by DLQI, EASI, and pruritus‐NRS scores across study visits. Additionally, the proportion of AD patients achieving EASI ≤ 7, EASI‐75, EASI‐90, DLQI 0/1, and pruritus NRS 0/1 increased greatly during the study period. The safety profile of upadacitinib‐based treatment strategies in the study was favorable, indicating that the drug is potentially safe and well tolerated. Altogether, the findings of this study support the conclusion that upadacitinib may have a favorable benefit‐risk profile in real‐world settings among patients with moderate‐to‐severe AD in SA. Longer‐term data on greater cohorts would be essential to further support the clinical utility and safety of upadacitinib‐based treatment strategies among AD patients in real‐life clinical practice.

## Author Contributions

Conceptualization, Shawky E. L. Saaty; investigation, Shawky E. L. Saaty, Hamza Salim Alshehri, Osama Alsharif, Azhar Alali, Mohamad Ibrahim Fatani, Abdulmajeed Alajlan, and Kamal Ahmed; data curation, Shereen Hammad, Tharwat Hamad, Ali Anwar, and Ibrahim Elnahal; methodology, Shawky E. L. Saaty, Hamza Salim Alshehri, Osama Alsharif, Azhar Alali, Mohamad Ibrahim Fatani, Abdulmajeed Alajlan, Shereen Hammad, Tharwat Hamad, Ali Anwar, Ibrahim Elnahal, and Kamal Ahmed, project administration, Shereen Hammad, Tharwat Hamad, Ali Anwar, and Ibrahim Elnahal; formal analysis, Kamal Ahmed and Shawky E. L. Saaty; supervision, Kamal Ahmed; writing–review and editing, Kamal Ahmed and Shawky E. L. Saaty.

## Funding

This research was funded by AbbVie.

## Disclosure

AbbVie participated in the study design, research, analyses, data collection, interpretation, review, and approval of the publication. The study sponsor had no role in clinical decision‐making for individual patients. No honoraria or payments were made for authorship. All authors had access to relevant data and participated in the drafting, review, and approval of this publication. All authors have read and agreed to the published version of the manuscript.

## Ethics Statement

The study was conducted according to the guidelines of the Declaration of Helsinki and approved by the Institutional Review Board (IRB) of KSA: East Jeddah Hospital, Jeddah, Jeddah Health Affairs‐IRB, 18‐July‐2022, A01398; KSA: Aseer Central Hospital, Aseer, Aseer Health Affairs‐IRB, 26‐Dec‐2022, REC‐06‐11‐2022; KSA: King Fahad General Hospital, Madinah, Madinah Health Affairs‐IRB, 05‐Mar‐2023, 22‐019; KSA: Hera General Hospital, Makkah, Makkah Health Affairs‐IRB, 03‐Oct‐2022, H‐02‐K‐076‐0922‐800; KSA: King Saud University Medical City, Riyadh, King Saud University‐IRB, 26‐Jan‐2023, E‐22‐7437; and KSA: Mohammad Dossary Hospital, Al Khobar, King Fahad Medical City‐IRB, 31‐Aug‐2022, 22‐385E2.

## Conflicts of Interest

Shawky E. L. Saaty, Hamza Salim Alshehri, Osama Alsharif, Azhar Alali, Mohamad Ibrahim Fatani, Abdulmajeed Alajlan, and Kamal Ahmed declare that they have no conflicts of interest, apart from their roles as investigators in an AbbVie‐funded study.

Shereen Hammad, Tharwat Hamad, Ali Anwar, and Ibrahim Elnahal are employees of AbbVie and may hold company stock or stock options.

## Supporting Information

Additional supporting information can be found online in the Supporting Information section.

## Supporting information


**Supporting Information** Table Supporting 1. Previous and current concomitant AD medications. Table Supporting 2. Previous and current concomitant non‐AD medications.

## Data Availability

All data generated are presented in the main manuscript and supporting files. Also, the data obtained and/or analyzed during this study are available on request from the corresponding author.
