# Supplementary material for: Real‐World Evidence of Upadacitinib Effectiveness in Moderate to Severe Atopic Dermatitis Patients in Saudi Arabia: A Retrospective Chart Review
Source: Dermatol Res Pract. 2026 Jul 15;2026:1238540. doi: 10.1155/drp/1238540 (PMC13373528; doi:10.1155/drp/1238540)
Supplement: Supplementary file 1 — Supporting Information Table Supporting 1. Previous and current concomitant AD medications. Table Supporting 2. Previous and current concomitant non‐AD medications. [file DRP-2026-1238540-s001.docx]

**Supplementary Material**

**Supplementary Tables**

**Table Supplementary 1.** Previous and Current Concomitant AD Medications (n = 109)

| **Variable** | **Baseline,  n (%)** | **3 Months,  n (%)** | **6 Months,  n (%)** |
| --- | --- | --- | --- |
| **Previously prescribed any medications for AD**  Yes  No | 90 (82.6%)  19 (17.4%) | 28 (25.7%)  81 (74.3%) | 20 (18.3%)  89 (81.7%) |
| **Patients received topical treatments**  **Patents received systemic treatments** | 25 (23%)  84 (77%) | 21 (19.3%)  88 (80.7%) | 13 (12%)  96 (88%) |
| **Previously assigned AD Medication***  Amoxicillin+ clavulanic acid  Azathioprine  Baricitinib  Betamethasone  Betamethasone+ Miconazole  Bilastine  Cetirizine  Chlorpheniramine maleate  Clobetasol  Cyclosporine  Desloratadine  Dupilumab  Fexofenadine  Fusidic acid  Fusidic acid+ Betamethasone  Gabapentin  Hydrocortisone  Hydroxyzine  Levocetirizine  Loratadine  Methotrexate  Methylprednisolone  Mometasone  Mometasone+ Miconazole  Mycophenolate mofetil  Omalizumab  Petrolatum Mineral Oil  Phototherapy  Pimecrolimus  Prednisolone  Promethazine  Rupatadine  Salicylic Acid  Tacrolimus  Tofacitinib  Urea  Vaseline | 0 (0%)  12 (5.5%)  3 (1.4%)  23 (10.5%)  1 (0.5%)  0 (0%)  13 (5.9%)  0 (0%)  6 (2.7%)  37 (16.8%)  3 (1.4%)  19 (8.6%)  1 (0.5%)  1 (0.5%)  4 (1.8%)  2 (0.9%)  7 (3.2%)  1 (0.5%)  1 (0.5%)  10 (4.5%)  4 (1.8%)  1 (0.5%)  9 (4.1%)  0 (0%)  1 (0.5%)  1 (0.5%)  2 (0.9%)  1 (0.5%)  5 (2.3%)  33 (15%)  1 (0.5%)  0 (0%)  1 (0.5%)  10 (4.5%)  1 (0.5%)  4 (1.8%)  2 (0.9%) | 1 (1.9%)  0 (0%)  0 (0%)  3 (5.7%)  3 (5.7%)  2 (3.8%)  8 (15.1%)  1 (1.9%)  2 (3.8%)  3 (5.7%)  1 (1.9%)  0 (0%)  0 (0%)  1 (1.9%)  2 (3.8%)  0 (0%)  1 (1.9%)  0 (0%)  1 (1.9%)  2 (3.8%)  0 (0%)  0 (0%)  13 (24.5%)  0 (0%)  0 (0%)  0 (0%)  0 (0%)  0 (0%)  0 (0%)  3 (5.7%)  0 (0%)  3 (5.7%)  0 (0%)  2 (3.8%)  0 (0%)  0 (0%)  1 (1.9%) | 0 (0%)  0 (0%)  0 (0%)  4 (12.1%)  1 (3%)  1 (3%)  4 (12.1%)  2 (6.1%)  1 (3%)  1 (3%)  1 (3%)  0 (0%)  1 (3%)  1 (3%)  0 (0%)  0 (0%)  0 (0%)  1 (3%)  0 (0%)  1 (3%)  0 (0%)  0 (0%)  5 (15.2%)  1 (3%)  0 (0%)  0 (0%)  0 (0%)  0 (0%)  0 (0%)  1 (3%)  0 (0%)  1 (3%)  0 (0%)  3 (9.1%)  0 (0%)  0 (0%)  3 (9.1%) |
| **Currently Prescribed any medication for AD other than Upadacitinib**  Yes  No | 84 (77.1%)  25 (22.9%) | 82 (75.2%)  27 (24.8%) | 82 (75.2%)  27 (24.8%) |
| **Patients received topical treatments**  **Patents received systemic treatments** | 79 (72.5%)  30 (27.5%) | 76 (69.7%)  33 (30.3%) | 75 (68.8%)  34 (34.3%) |
| **Currently assigned AD Medication other than Upadacitinib***  Amoxicillin+ Clavulanic acid  Betamethasone  Betamethasone+ Miconazole  Bilastine  Calcipotriol+ Betamethasone  Cetirizine  Chlorpheniramine Maleate  Clobetasol  Cyclosporine  Desloratadine  Dexpanthenol  Dupilumab  Fluocinolone / Hydroquinone / Tretinoin  Fexofenadine  Fusidic acid  Fusidic acid +Betamethasone  Hydrocortisone  Hydroxyzine  Levocetirizine  Loratadine  Mometasone  Mometasone+ Miconazole  Petrolatum Oil  Pimecrolimus  Prednisolone  Rupatadine  Tacrolimus  Urea  Vaseline  White paraffin | 1 (0.5%)  11 (5.7%)  3 (1.6%)  2 (1.1%)  1 (0.5%)  48 (25.1%)  6 (3.1%)  3 (1.6%)  4 (2.1%)  1 (0.5%)  0 (0%)  0 (0%)  0 (0%)  1 (0.5%)  5 (2.6%)  4 (2.1%)  3 (1.6%)  1 (0.5%)  1 (0.5%)  2 (1.1%)  38 (19.9%)  0 (0%)  2 (1.1%)  3 (1.6%)  4 (2.1%)  3 (1.6%)  8 (4.2%)  4 (2.1%)  10 (5.2%)  22 (11.5%) | 0 (0%)  13 (7.7%)  1 (0.6%)  1 (0.6%)  1 (0.6%)  44 (25.9%)  5 (2.9%)  1 (0.6%)  1 (0.6%)  1 (0.6%)  0 (0%)  0 (0%)  0 (0%)  1 (0.6%)  4 (2.4%)  2 (1.2%)  2 (1.2%)  1 (0.6%)  1 (0.6%)  5 (2.9%)  32 (18.8%)  1 (0.6%)  2 (1.2%)  4 (2.4%)  1 (0.6%)  1 (0.6%)  7 (4.1%)  5 (2.9%)  10 (5.6%)  23 (13.5%) | 1 (0.6%)  15 (8.6%)  0 (0%)  2 (1.1%)  1 (0.6%)  41 (23.6%)  3 (1.7%)  0 (0%)  1 (0.6%)  2 (1.1%)  1 (0.6%)  1 (0.6%)  1 (0.6%)  0 (0%)  4 (2.3%)  5 (2.9%)  5 (2.9%)  0 (0%)  2 (1.1%)  6 (3.4%)  30 (17.2%)  3 (1.7%)  3 (1.7%)  6 (3.4%)  0 (0%)  1 (0.6%)  4 (2.3%)  6 (3.4%)  7 (4.1%)  23 (13.2%) |

Abbreviations: AD, Atopic dermatitis.

*Patient might receive more than one medication

**Table Supplementary 2.** Previous and Current concomitant non-AD Medications (n = 109)

| **Variable** | **Baseline,  n (%)** | **3 Months,  n (%)** | **6 Months,  n (%)** |
| --- | --- | --- | --- |
| **Prescribed any concomitant non-AD medication**  Yes  No  NA | 17(15.5%)  43 (39.4%)  49 (45%) | 12 (11%)  54 (49.5%)  43 (39.4%) | 10 (9.2%)  59 (54.1%)  40 (36.7%) |
| **Concomitant non- AD medication***  Acyclovir  Abatacept  Amitriptyline  Amlodipine  Atorvastatin  Azithromycin  Bisoprolol  Budesonide  Budesonide/Formoterol  Calcipotriene/Betamethasone  Carboxymethylcellulose  Cephalexin  Cholecalciferol  Cyclopentolate  Doxycycline  Desloratadine  Ferrous sulphate  Fluconazole  Fluticasone/Salmeterol  Gliclazide  Hydrochlorothiazide  Hydroxychloroquine  Indapamide  Insulin glargine  Isotretinoin  Levothyroxine  Lornoxicam  Metformin  Methotrexate  Minoxidil  Moxifloxacin  Omeprazole  Perindopril Arginine  Polyethylene glycol+ Propylene glycol  Salbutamol  Selenium  Sodium Hyaluronate  Tobramycin+ Dexamethasone  Valsartan  Vitamin D | 0 (0%)  1 (3.03%)  1 (3.03%)  1 (3.03%)  1 (3.03%)  1 (3.03%)  1 (3.03%)  1 (3.03%)  1 (3.03%)  0 (0%)  0 (0%)  1 (3.03%)  1 (3.03%)  0 (0%)  1 (3.03%)  1 (3.03%)  2 (6.06%)  1 (3.03%)  1 (3.03%)  0 (0%)  1 (3.03%)  1 (3.03%)  1 (3.03%)  1 (3.03%)  0 (0%)  2 (6.06%)  1 (3.03%)  1 (3.03%)  1 (3.03%)  1 (3.03%)  0 (0%)  1 (3.03%)  1 (3.03%)  1 (3.03%)  2 (6.06%)  1 (3.03%)  0 (0%)  0 (0%)  1 (3.03%)  0 (0%)  1 (3.03%) | 1 (3.01%)  1 (3.01%)  1 (3.01%)  1 (3.01%)  1 (3.01%)  0 (0%)  1 (3.01%)  1 (3.01%)  1 (3.01%)  0 (0%)  1 (3.01%)  0 (0%)  1 (3.01%)  1 (3.01%)  1 (3.01%)  0 (0%)  1 (3.01%)  0 (0%)  1 (3.01%)  1 (3.01%)  1 (3.01%)  0 (0%)  1 (3.01%)  1 (3.01%)  1 (3.01%)  2 (6.25%)  0 (0%)  1 (3.01%)  0 (0%)  1 (3.01%)  1 (3.01%)  1 (3.01%)  1 (3.01%)  1 (3.01%)  2 (6.25%)  1 (3.01%)  0 (0%)  0 (0%)  1(3.01%)  1(3.01%) | 1 (2.5%)  0 (0%)  1 (2.5%)  1 (2.5%)  2 (5.0%)  0 (0%)  1 (2.5%)  1 (2.5%)  1 (2.5%)  1 (2.5%)  0 (0%)  0 (0%)  2 (5.0%)  0 (0%)  1 (2.5%)  0 (0%)  0 (0%)  0 (0%)  1 (2.5%)  1 (2.5%)  1 (2.5%)  0 (0%)  1 (2.5%)  1 (2.5%)  1 (2.5%)  2 (5.0%)  0 (0%)  1 (2.5%)  0 (0%)  1 (2.5%)  0 (0%)  1 (2.5%)  1 (2.5%)  1 (2.5%)  2 (5.0%)  1 (2.5%)  1 (2.5%)  1 (2.5%)  1 (2.5%)  1 (2.5%) |
| **Indications for concomitant non- AD medication***  Allergy  Androgenic Alopecia  Bacterial infection  Bronchial asthma  Conjunctivitis  Corneal Edema & Ulceration  Dandruff  Depression  Diabetes mellitus  Eye dryness  Eczema Boils  Fungal infection  Gastrointestinal reflux  Hyperlipidemia  Hypertension  Hypothyroidism  Psoriasis  Inflammation  Iron deficiency anemia  Rheumatoid arthritis  Vitamin D Deficiency | 1 (2.9%)  1 (2.9%)  2 (5.9%)  5 (14.7%)  1 (2.9%)  0 (0%)  1 (2.9%)  1 (2.9%)  2 (5.9%)  0 (0%)  0 (0%)  2 (5.9%)  1 (2.9%)  1 (2.9%)  5 (14.7%)  2 (5.9%)  0 (0%)  1 (2.9%)  2 (5.9%)  4 (11.8%)  1 (2.9%) | 0 (0%)  1 (2.9%)  0 (0%)  5 (14.3%)  1 (2.9%)  4 (11.4%)  1 (2.9%)  1 (2.9%)  3 (8.6%)  0 (0%)  2 (5.7%)  0 (0%)  1 (2.9%)  1 (2.9%)  6 (17.1%)  2 (5.7%)  0 (0%)  0 (0%)  1 (2.9%)  0 (0%)  2 (5.7%) | 0 (0%)  1 (2.7%)  0 (0%)  5 (13.5%)  1 (2.7%)  2 (5.4%)  1 (2.7%)  1 (2.7%)  3 (8.1%)  1 (2.7%)  0 (0%)  0 (0%)  1 (2.7%)  2 (5.4%)  6 (16.2%)  2 (5.4%)  1 (2.7%)  0 (0%)  0 (0%)  0 (0%)  3 (8.1%) |
| **Ongoing status of concomitant non-AD medication**  Yes  No | 21 (65.6%)  11 (34.3%) | 23 (65.7%)  12 (34.3%) | 27 (72.9%)  10 (8.1%) |

Abbreviations: AD, Atopic dermatitis.

*Patient might suffer from more than one medical condition.
